# Training Pigs for Oral Glucose Tolerance Test—Six Years’ Experience of a Refined Model

**DOI:** 10.3390/ani11061677

**Published:** 2021-06-04

**Authors:** Elin Manell, Patricia Hedenqvist, Marianne Jensen-Waern

**Affiliations:** Department of Clinical Sciences, Swedish University of Agricultural Sciences, 75007 Uppsala, Sweden; patricia.hedenqvist@slu.se (P.H.); marianne.jensen-waern@slu.se (M.J.-W.)

**Keywords:** pig, bottle-feeding, oral glucose tolerance test, training

## Abstract

**Simple Summary:**

Animal models for human diseases are used in situations where studies cannot be carried out on humans. While animal models in biomedical research play a pivotal part in the development of new and safe treatments for humans, it is important that the animals are used in the best way and that possible refinements are considered. Pigs are often used to model humans since the two species share many anatomical and physiological characteristics. This publication describes refinements in the training technique of pigs prior to an oral glucose tolerance test, a test commonly used in diabetes research where the individual drinks a certain amount of glucose followed by blood sampling. Sharing these results with the research community will help other researchers to successfully train pigs in such studies.

**Abstract:**

Animal models of human diseases are important in biomedical research. When using animals for scientific purposes, the 3Rs (replace, reduce, refine) should be considered. Refinement of animal models is essential to ensure best use of animals, which is important for ethical reasons and to retrieve reliable research data. The present publication describes improvements to an oral glucose tolerance test (OGTT) model for pigs published in 2016. Historical data from 42 pigs were used to describe improvements in the training technique over six years. Pigs of various breeds and ages can be trained to bottle-feed glucose dissolved in water to undergo OGTT. This publication describes different tips and techniques to apply for successful training and will help researchers to minimize exclusions of pigs due to unsuccessful training. The improvements are an important contribution to the 3Rs.

## 1. Introduction

In biomedical research, animal models for human diseases play a pivotal part in finding new or improved treatments. When using animals in research, the 3Rs, replace, reduce and refine, should be considered [1]. It is important to refine animal models and to disseminate results to ensure that all animals are used in the best possible way to minimise negative effects on the animals, reduce numbers of animals needed, and to acquire research data with high validity. Pigs are used as large animal models since they share many anatomical and physiological characteristics with humans [2]. Training the pigs before they are used in research studies is always important to minimise negative impact on animal welfare, since stressful situations can induce behavioural responses such as a flight response or vocalisations, which are indicators of fear [3]. Repeated negative experiences can induce chronic stress with a large impact on the physiology and welfare of the animals [4]. Furthermore, stress responses will affect a range of different physiological parameters, which could affect research results [3]. For example, blood glucose concentrations increase rapidly in response to acute stress [5] and can bias studies on diabetes and metabolism. 

In diabetes research, the oral glucose tolerance test (OGTT) is widely used to diagnose diabetes and to evaluate effects of treatments. In 2016, we published a refined model for OGTT in pigs that reduced experimental variability compared to the previously described method [6]. With the refined method, pigs are trained to voluntarily drink glucose solution from a feeding bottle. Except for our publication, there is no scientific literature on how to train pigs to bottle-feed an oral glucose load. We now have six years of experience in training pigs for OGTT. As decisions in laboratory animal medicine should be based on science and proven experience, the aim of the present publication is to describe improvements in the training techniques that were developed over time, and to describe the application of the refined OGTT model for pigs of different breeds and ages.

## 2. Materials and Methods

In the present publication, historical data from medical records was used to describe experiences and improvements of the refined OGTT model previously described [6]. All pigs were used in research studies with ethics permits granted from the Uppsala Committee of Animal Research Ethics for the respective study. No animals were acquired for the present publication.

Between 2014 and 2020, 42 pigs were trained to bottle-feed glucose dissolved in water at the Department of Clinical Sciences, Swedish University of Agricultural Sciences. Animal characteristics are presented in Table 1. High health SPF [7] domestic pigs (Yorkshire, Swedish Landrace, and/or Hampshire) were acquired from the university herd (Swedish Livestock Research Centre, Lövsta, SLU). Göttingen minipigs were acquired from Ellegaard, Dalmose, Denmark. All pigs were housed at the department of Clinical Sciences in individual pens (3 m^2^) within sight and sound of one another. Straw and wood shavings were used for bedding. A 10:14 h light/dark schedule (lights on at 6 a.m.) was applied, and an infrared lamp was provided in a corner of each pen for growing pigs. The room temperature was 16–18 °C. Domestic pigs were fed commercial finisher diet (Solo 330, Lantmännen, Sweden) twice daily, the amount depending on body weight, according to the Swedish University of Agricultural Sciences regimen for growing pigs [8]. Minipigs were fed minipig diet (SMP (E) SQC, Special Diet Services, England) twice daily in an amount according to the feeding guide from the manufacturer. Water was provided ad libitum.

All pigs were allowed a two-week acclimatisation period before the initiation of the studies. During this time, the animals were socialised and trained to handing and clinical examination, and to step onto an electronic scale. The pigs were also trained to drink glucose dissolved in water from a feeding bottle. Details of the training are reported in the following section. Perfect bottle feeding was defined as actively suckling the entire content of the bottle without any intermission or spill. 

## 3. Results and Discussion

**Figure 1:**
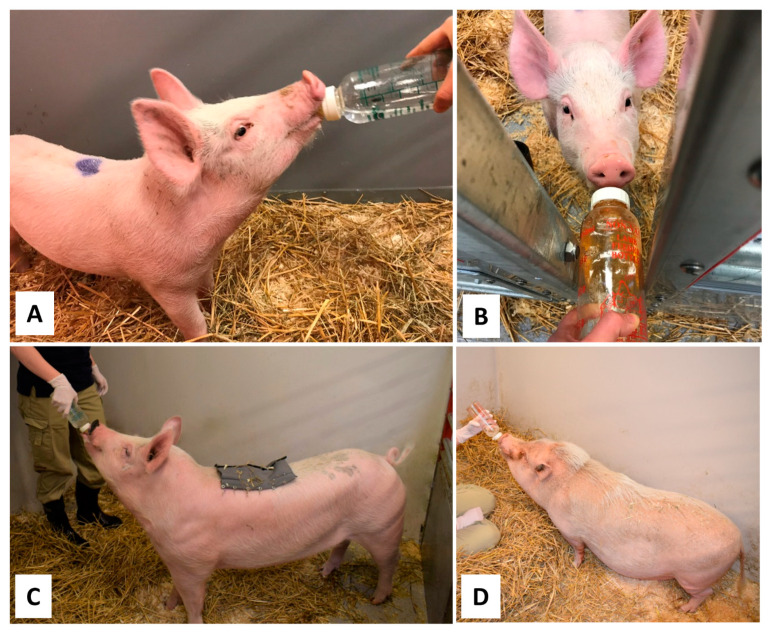
Bottle-feeding training of pigs prior to the oral glucose tolerance test. (**A**) Eight-week-old domestic pig, trained with glucose dissolved in water. (**B**) Eight-week-old domestic pig, trained with apple juice through the front of the pen. (**C**) Five-month-old domestic pig, trained with glucose dissolved in water. (**D**) Two-year-old Göttingen minipig, trained with glucose dissolved in water.

We have successfully trained both young domestic pigs (<6 months old, up to 120 kg) and adult Göttingen minipigs (2–3 years old) to bottle-feed an oral glucose load (Glukos APL Pulver till oral lösning, APL, Stockholm, Sweden), as shown in Figure 1. Pigs are curious and very investigative when it comes to unfamiliar objects. This is an advantage for getting the pigs interested in the feeding-bottle. On the other hand, some pigs are so curious about the person entering the pen that it can be difficult to get the pig to focus on the feeding bottle. In such instances, it is better to start bottle-feeding training through the front of the pen if possible, depending on the construction of the pen (Figure 1B). Once the pig has learnt an efficient bottle-feeding technique and become interested in the content of the bottle, it is possible to enter the pen for bottle feeding. However, if needed, OGTT can also be carried out by bottle feeding through the front of the pen. 

In the original publication [6], pigs were trained to bottle-feed glucose dissolved in water, or if the pigs were hesitant to glucose, broth was used, and glucose content gradually increased while broth content was decreased. Some pigs refuse to drink both glucose and broth. However, we learned that apple juice is a good option for training the technique of efficient bottle-feeding, which can then be gradually replaced by glucose dissolved in water. For training purposes, caster sugar can be used instead of glucose. The concentration of the glucose/sugar solution used for training and maintenance can be lower than the concentration used for OGTT. A common concentration used in our facility is 0.15 g/mL, of which the pigs would receive 100 mL during a training session. For a 30 kg pig, that corresponds to 0.5 g/kg BW.

**Figure 2:**
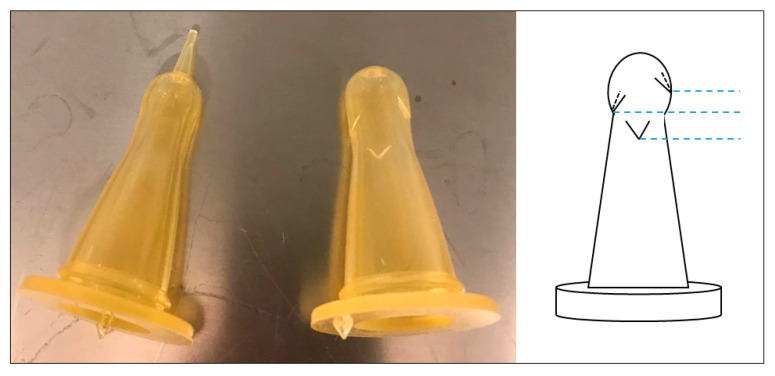
Picture and schematic drawing of modified rubber teat for feeding-bottle used for oral glucose tolerance testing in pigs. Blue dotted lines demonstrate three different levels on the teat at which cuts are made.

For bottle feeding, modified rubber teats for lambs (Anti-Vac teat for lamb feed bottle, Kerbl, Buchbach, Germany) were used (Figure 2). The holes in the teats need to be big enough for the pig not to lose interest due to low flow of liquid, but small enough for the animal not to spill. We found that cutting three extra holes with a pair of scissors accomplishes this and worked well with both domestic pigs and Göttingen minipigs. The cuts were made on three different levels to minimise risk for damage due to weakness on one level (Figure 2).

In the original publication [6], it was stated that pigs should be bottle-fed every day to not lose the ability to bottle-feed. However, with experience we have discovered that once the pigs have learnt a perfect bottle-feeding technique and learnt to like glucose/sugar, they do not need to be bottle-fed every day. In fact, domestic pigs have been left for a month without bottle feeding and could still bottle-feed perfectly. We also found that some pigs require more than two weeks to learn a perfect bottle-feeding technique (up to 4–5 weeks). A two-week training period is standard at our facility, but to be able to include more pigs in OGTT interventions, a longer training period may be necessary. A few pigs do not learn to bottle-feed even within 5 weeks, and might never learn, which must be taken into account when deciding how many pigs to procure.

Four minipigs were trained to bottle-feed caster sugar dissolved in water every day for seven consecutive days. All four were interested in the sugar solution from the start but improved their technique until they consumed the content without any spill. After having acquired a perfect technique for bottle feeding, the next two training sessions were two and three weeks later. Despite this gap, the pigs could still bottle-feed perfectly. In the following 4.5 months, the minipigs were not bottle-fed and then needed 1–2 training sessions until they bottle-fed perfectly again. Although all training was carried out with caster sugar dissolved in water, the minipigs consumed 75 g glucose (Glukos APL Pulver till oral lösning, APL, Stockholm, Sweden) dissolved in 250 mL water within 40–87 s. There have not been any juvenile minipigs housed at our department during the last six years. Consequently, since no animals were acquired for this publication, we do not have any data for applying the refined OGTT model on juvenile minipigs, however it is plausible to assume that juvenile minipigs also can be trained to bottle-feed glucose dissolved in water. In the original publication of the OGTT model [6], it was pointed out that bottle feeding should start at a young age. This was based on data from domestic pigs, which are usually used for scientific purposes when they are young. Minipigs are often used at various ages, and we found it easy to train adult minipigs (2–3 years old) to bottle-feed glucose solution.

## 4. Conclusions

Pigs of various breeds and ages can be trained to bottle-feed glucose dissolved in water to undergo OGTT. Based on six years of experience, we recommend the following:

The improvements described in this publication are an important refinement contributing to better use of porcine models in research.

(1) Initial bottle-feeding training should be carried out every day until the individual pig has learnt a perfect bottle-feeding technique.
(2) Pigs hesitant to drink glucose dissolved in water can be trained with broth or apple juice and later become accustomed to glucose by gradual replacement.
(3) For pigs that are more curious about the person in the pen than of the feeding bottle, initial training can be carried out through the front of the pen.
(4) When using rubber teats for lambs, cut three extra holes at different levels for efficient speed of bottle feeding.
(5) Pigs can be trained with caster sugar instead of glucose.
(6) For training and maintenance, low glucose/sugar concentrations (~0.15g/mL) can be used.
(7) Most pigs learn to bottle-feed within two weeks, but a longer training period, up to 4–5 weeks, can be applied if more pigs must undergo OGTT intervention.
(8) A few pigs may never learn to bottle-feed, which should be taken into account when deciding how many pigs to procure.
(9) Pigs that have learnt to bottle-feed perfectly do not have to be trained every day; they can be left for at least a month.

## Figures and Tables

**Table 1:** Characteristics of study animals.

Breed	Sex (No. Females/Males)	Age at Start of Training(Range)
Domestic pigs (Yorkshire, Swedish Landrace, and/or Hampshire)	17/21	5–7 weeks
Göttingen minipigs	4/0	25–32 months

## Data Availability

The data presented in this study are available on request from the corresponding author.
